# Analysis of phase III clinical trials in metastatic NSCLC to assess the correlation between QoL results and survival outcomes

**DOI:** 10.1186/s12916-023-02953-0

**Published:** 2023-07-03

**Authors:** Alberto Servetto, Massimo Di Maio, Fabio Salomone, Fabiana Napolitano, Chiara Paratore, Fabrizio Di Costanzo, Giuseppe Viscardi, Antonio Santaniello, Luigi Formisano, Roberto Bianco

**Affiliations:** 1 Department of Clinical Medicine and Surgery, University of Naples Federico II, Via Sergio Pansini 5, Naples, Italy; 2 Department of Oncology, University of Turin, Division of Medical Oncology, Ordine Mauriziano Hospital, Turin, Italy; 3 Department of Oncology, ASL TO4, Ivrea Community Hospital, Ivrea, Italy; 4 Department of Pneumology and Oncology, AORN Ospedali Dei Colli-Monaldi, Naples, Italy

**Keywords:** QoL, Quality of life, Metastatic NSCLC, Lung cancer, PROs, Patient reported outcomes, Randomized controlled trials, RCTs

## Abstract

**Background:**

In addition to improving survival outcomes, new oncology treatments should lead to amelioration of patients’ quality of life (QoL). Herein, we examined whether QoL results correlated with PFS and OS outcomes in phase III randomized controlled trials (RCTs) investigating new systemic treatments in metastatic non-small cell lung cancer (NSCLC).

**Methods:**

The systematic search of PubMed was conducted in October 2022. We identified 81 RCTs testing novel drugs in metastatic NSCLC and published in the English language in a PubMed-indexed journal between 2012 and 2021. Only trials reporting QoL results and at least one survival outcome between OS and PFS were selected. For each RCT, we assessed whether global QoL was “superior,” “inferior,” or with “non-statistically significant difference” in the experimental arm compared to the control arm.

**Results:**

Experimental treatments led to superior QoL in 30 (37.0%) RCTs and inferior QoL in 3 (3.7%) RCTs. In the remaining 48 (59.3%) RCTs, a statistically significant difference between the experimental and control arms was not found. Of note, we found a statistically significant association between QoL and PFS improvements (*X*^2^ = 3.93, *p* = 0.0473). In more detail, this association was not significant in trials testing immunotherapy or chemotherapy. On the contrary, in RCTs testing target therapies, QoL results positively correlated with PFS outcomes (*p* = 0.0196). This association was even stronger in the 32 trials testing EGFR or ALK inhibitors (*p* = 0.0077). On the other hand, QoL results did not positively correlate with OS outcomes (*X*^2^ = 0.81, *p* = 0.368). Furthermore, we found that experimental treatments led to superior QoL in 27/57 (47.4%) trials with positive results and in 3/24 (12.5%) RCTs with negative results (*p* = 0.0028). Finally, we analyzed how QoL data were described in publications of RCTs in which QoL outcomes were not improved (*n* = 51). We found that a favorable description of QoL results was associated with sponsorship by industries (*p* = 0.0232).

**Conclusions:**

Our study reveals a positive association of QoL results with PFS outcomes in RCTs testing novel treatments in metastatic NSCLC. This association is particularly evident for target therapies. These findings further emphasize the relevance of an accurate assessment of QoL in RCTs in NSCLC.

**Supplementary Information:**

The online version contains supplementary material available at 10.1186/s12916-023-02953-0.

## Background

Overall survival (OS) and progression-free survival (PFS) are the primary study endpoints in randomized controlled trials (RCTs) testing novel treatments in oncology [1]. Particularly, the assessment of OS is commonly recognized as the best instrument to measure the efficacy of novel therapeutic strategies, compared to standard available treatments. PFS improvements, although suggestive of clinical efficacy of anticancer treatments, do not necessarily translate into OS advantage [2–4]. Hence, PFS should be considered solely as a surrogate estimate of efficacy. However, despite this awareness among clinicians and researchers, some drugs have received regulatory approval based on improvements in PFS, non-reinforced by positive OS results [5].

In addition to increasing OS, the aim of new treatments should be to improve the quality of life (QoL) of patients with cancer [6]. In recent years, the oncology community devoted more attention to the assessment of QoL outcomes in RCTs as well as in routine clinical practice. Indeed, this recognition led to the publication of guidelines by the European Society for Medical Oncology (ESMO) for a comprehensive assessment of patient-reported outcomes (PROs) in clinical practice [7]. Furthermore, the ESMO Magnitude of Clinical Benefit Scale (ESMO-MBCS), developed to measure thoroughly the weight of clinical benefit of new anticancer therapies, assigns positive scores for treatments that demonstrate improvement in QoL [8, 9], thus encouraging their use with a stronger recommendation.

In patients with cancer, QoL is certainly influenced by symptoms caused by disease burden, side effects of therapies, and daily life domains, such as emotional, physical, and social functions. However, although the development of new anticancer treatments aims to prolong the survival of patients, such progresses are not always associated with improvements in QoL. Previous studies analyzed data from RCTs in oncology and revealed that, for systemic anticancer treatments, improvements in PFS did not correlate with QoL benefits [10, 11]. More recently, by investigating a selected list of RCTs in oncology published in 2019, Samuel and colleagues found a positive correlation between improvements in OS and QoL benefits for experimental cancer therapies [12]. For patients, as well as medical oncologists, understanding the potential benefits of treatments in terms of both survival and QoL is crucial to take a shared decision regarding the most appropriate therapeutic approach. Of note, previous studies revealed that integration of patient-reported symptom monitoring in clinical practice might promote benefits on both global QoL and OS [13].

Assessment of QoL is particularly relevant in patients with metastatic non-small cell lung cancer (NSCLC), due to symptoms that have a strong negative impact on QoL, such as dyspnea, cough, hemoptysis, shortness of breath, pain, and possible neurological symptoms [14]. Furthermore, because of disease extent and treatment-related adverse events, in addition to the aforementioned symptoms, these patients frequently experience deterioration of psychological dimension, characterized by anxiety, depression, and malnutrition [15]. In recent years, the treatment landscape of patients with metastatic NSCLC has been revolutionized by immune checkpoint inhibitors (ICIs) and novel selective inhibitors for oncogene-addicted disease. In addition to better survival outcomes, some of these novel treatments demonstrated improvements in QoL as well. However, it is unclear whether a correlation between QoL outcomes and survival outcomes exists and the extent of such correlation for various drug classes.

Despite the well-known relevance of QoL assessment in patients undergoing treatments for cancer, we and other groups have previously revealed that QoL results are poorly reported in publications of RCTs in oncology, hindering a complete evaluation of the effects of new anticancer drugs [16–19]. Furthermore, some publications of trial results describe QoL data with an inappropriate positive framing, overestimating the real benefits obtained with experimental treatments [12].

Herein, we report the results from our study aiming to investigate QoL outcomes in publications of phase III RCTs testing novel systemic treatments in patients with metastatic NSCLC, published between 2012 and December 2021. We selected phase III RCTs because they explore new therapies that may receive approval from regulatory agencies. In addition, RCTs should include QoL assessment among study endpoints. We evaluated whether QoL results correlate with PFS and OS outcomes, exploring such correlation in different drug classes. Finally, we analyzed how QoL results were described in these manuscripts and if favorable framing was enriched in profit studies, compared to non-profit trials.

## Methods

### Data source and search strategy

We analyzed publications of phase III RCTs investigating novel systemic therapies for the treatment of metastatic NSCLC, published between January 2012 and December 31, 2021. The following terms were searched in PubMed: “NSCLC” OR “non-small cell lung cancer” OR “non small cell lung cancer” OR “lung cancer.” The following PubMed filters were applied: “Clinical Trial, Phase III”, “Randomized controlled trials,” and “English Language.” The research string can be found in Additional file 1: Table S1. The PubMed database was interrogated in October 2022. All information about trials was collected from the article or through the https://www.clinicaltrials.gov website.

### Inclusion and exclusion criteria

The titles and abstracts were examined. Only phase III RCTs in metastatic NSCLC that reported QoL results and at least one survival outcome between OS and PFS were considered eligible for further analysis. The following RCTs were excluded from the analysis: (1) trials testing surgery and/or radiotherapy; (2) trials non-including QoL among study endpoints; (3) trials not reporting QoL results in primary or secondary publications; (4) trials not reporting results of at least one survival outcome between PFS and OS; (5) trials evaluating multiple schedules of administration of the same drug; (6) trials of supportive care or behavioral approaches; (7) not phase III RCTs; (8) trials in locally advanced, neo-adjuvant, or adjuvant settings; (9) study protocols; (10) subgroup or post hoc analysis of previously published trials; (11) brief reports or case studies; (12) research of screening methodologies; and (13) pooled analysis of multiple trials. The remaining full texts were downloaded for further analysis.

### Data collection

Data were extracted from publications and reported in an electronic database. These included first author, digital object identifier (DOI), name of the trial, journal, date of publication, and class of therapy investigated (chemotherapy, immunotherapy, or target therapy). Data were analyzed by two independent investigators. Discrepancies were resolved by consensus.

The following data were also extracted from trials: histological subtype (squamous cell carcinoma (SCC), non-squamous cell carcinoma (NSCC), “non-specified histology”); if required, mandatory mutations for patients enrolment (“*EGFR* mutations,” “*ALK* translocations,” or “*KRAS* mutations”); funding (“profit,” when the trial was sponsored by a pharma company, or “no profit,” when the trial was designed and conducted by academic institution/s); results (“positive,” when statistically significant advantage in the primary endpoint was reached in experimental arm over control arm, or “negative”, when primary endpoint was not met. For RCTs with PFS and OS as co-primary endpoints, results were considered positive only if both endpoints were met); study design (“superiority” when the aim of the trial was to detect an advantage in experimental drug over standard treatment or “non-inferiority” when the aim was to show a same efficacy between the experimental and the control arm); masking (“blinded,” when neither researchers nor patients were aware of the assigned treatment, or “open-label”); involved countries (“multi,” involving institutions of two or more different countries, or “single” when they belonged to a single country); and primary endpoints (OS, PFS, safety, QoL, and ORR).

Assessment of QoL in RCTs was investigated by examining the methods sections of the articles or study protocols. When protocols were not available as supplementary material of publications, they were searched on the https://www.clinicaltrials.gov/ website. We interrogated study protocols to find out whether QoL was assessed among endpoints (primary, secondary, exploratory endpoint, or non-analyzed) and the types of tests utilized in the trial to measure QoL.

For trials not reporting QoL results in primary publication, we assessed their disclosure in secondary manuscripts. Secondary articles were searched among the list of articles obtained with our research strategy (Additional file 1: Table S1). Only for one trial, a secondary manuscript with QoL results was published later than December 2021 [20]. We also searched potential secondary publications in PubMed using the name of the drug and the study’s acronym, but this research did not produce any additional results. Five trials of non-reporting QoL results in primary or secondary publications disclosed QoL data at international conferences and were included in the analysis. For trials reporting QoL results in both primary and secondary QoL-focused manuscripts, QoL data were extracted from the secondary articles, supposed to be more accurate.

For RCTs with non-available or immature OS data at the time of primary publication, we evaluated the final OS results reported in secondary publications.

### Data interpretation

Global QoL results were evaluated based on the comparison between the experimental arm and the control arm, as previously reported [12]. Hence, we did not consider comparisons of “before and after treatment” in each arm. Based on these premises, QoL outcomes were classified as follows: (1) “superior,” when a statistically significant improvement in global health status (GHS)/global QoL results were recorded in the experimental arm, compared to control arm; (2) “inferior,” when in the experimental arm, a statistically significant decline in GHS/global QoL was found, compared to control arm; (3) “no statistically significant difference” in terms of GHS/global QoL between the experimental and control arms. When only for some symptoms or some domains a statistically significant improvement was found, but with no improvement in GHS/global QoL in the trial, QoL outcomes were classified in the “no statistically significant difference” category.

We analyzed abstracts and full texts to assess how authors described and interpreted QoL results. The description was considered “favorable” when (i) authors claimed that global QoL “did not worsen” upon treatment, because a novel therapy should aim to guarantee an improvement in QoL, and not only a reduced worsening; (ii) authors emphasized the effect of treatment on QoL outcomes with superior scores only in some symptoms or functions but without a concomitant improvement in GSH/global QoL; and (iii) authors declared an improvement in QoL results in the same arm, comparing pre- and post-treatment outcomes, without an appropriate comparison between the two arms. The description of QoL data was considered “neutral” when the description and discussion of QoL results were coherent with the data reported in the manuscript or at international conferences. Two investigators independently assessed the description of QoL results in RCTs. Discrepancies were resolved by consensus.

### Statistical analysis

Statistical differences between the analyzed groups were calculated using the chi-square test or Fisher’s exact test in the Prism – GraphPad software v.9.

## Results

### Study characteristics

**Fig. 1:**
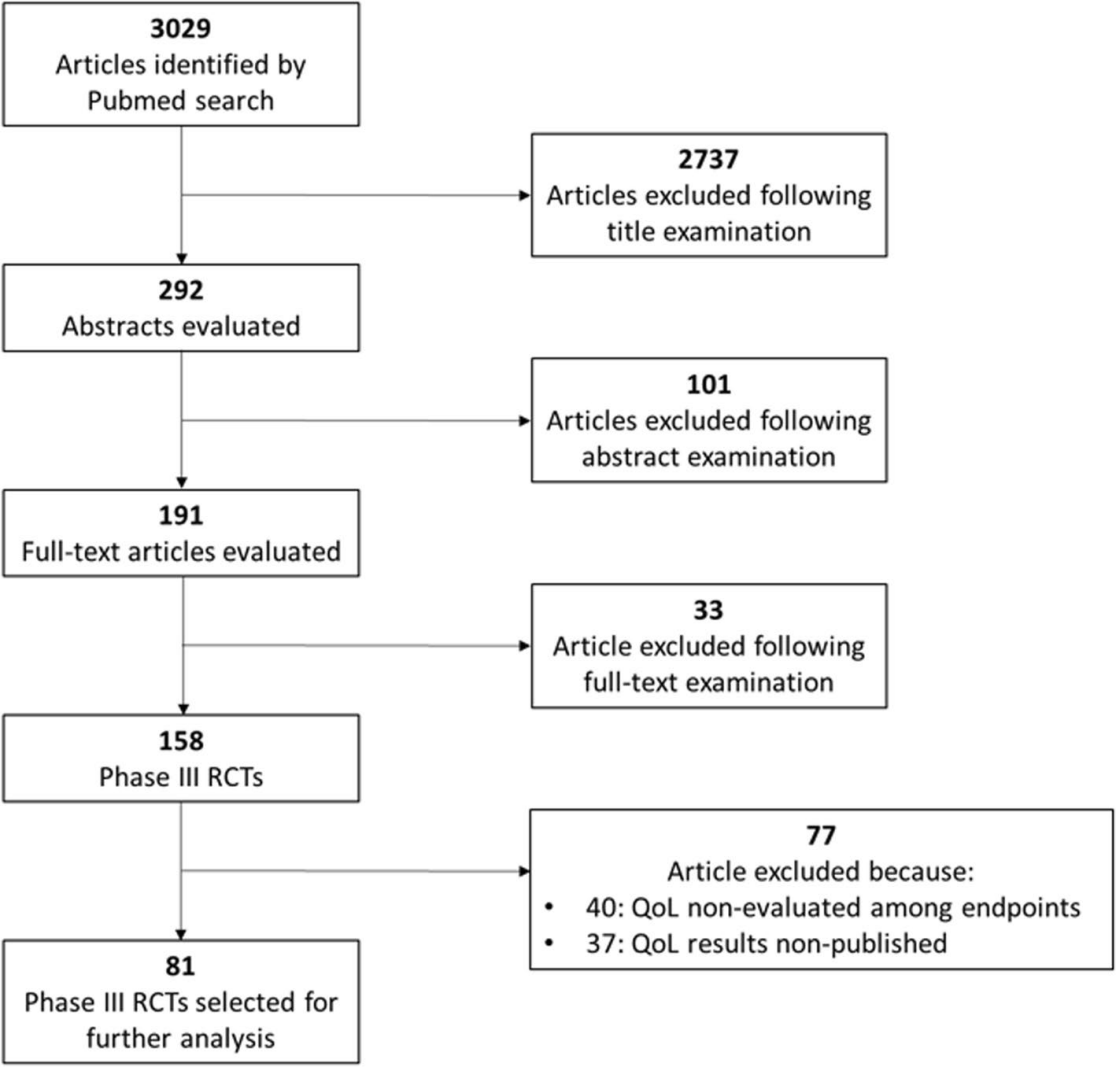
PRISMA flowchart diagram

Our PubMed research identified 3029 articles (Fig. 1). We found 158 phase III RCTs testing systemic treatments in patients with metastatic NSCLC, whose primary manuscript was published between January 2012 and December 2021. However, 40/158 (25.3%) trials were excluded because QoL was not evaluated among study endpoints. Furthermore, 37/158 (23.4%) RCTs were excluded because QoL results were published neither in primary nor in secondary publications. For none of these 37 trials, QoL data were presented at international conferences. Finally, 81 phase RCTs were selected for further analysis (Fig. 1). QoL results were reported in primary or secondary publications, in addition to PFS and/or OS results. Five RCTs reported QoL results at international conferences. The list of the 81 trials is reported in Additional file 1: Table S1. A summary of the characteristics of these studies is reported in Table 1. The 81 articles included in the analysis were published in 14 journals (*Annals of Oncology*, *British Journal of Cancer*, *Cancer*, *Cancer Cell*, *Clinical Lung Cancer*, *European Journal of Cancer*, *JAMA*, *JAMA Oncology*, *Journal of Clinical Oncology*, *Journal of Thoracic Oncology*, *The Lancet*, *Lancet Healthy Longevity*, *Lancet Oncology*, *Lung Cancer*, and *New England Journal of Medicine*). The highest percentage of trials were found in *Lancet Oncology* (25.9%, *n* = 21/81) and *New England Journal of Medicine* (18.5%, *n* = 15). Target therapy, chemotherapy, and immunotherapy were investigated in 50 (61.7%), 17 (21.0%), and 16 (19.8%) trials, respectively. Agents directed against EGFR, ALK, or other targets were tested in 24 (29.6%), 8 (9.9%), and 18 (22.2%) trials, respectively. In 49 (60.5%) studies, patients were recruited regardless of NSCLC histology. However, for the 12 trials enrolling patients with non-specified NSCLC histology but with mandatory presence of EGFR mutations (*n* = 6), ALK rearrangements (*n* = 5), or KRAS mutations (*n* = 1), the vast majority of enrolled patients (> 90% for each study) had a diagnosis of lung adenocarcinoma. Trials enrolling exclusively patients with non-squamous cell carcinoma (NCSS) or squamous cell carcinoma (SCC) were 25 (30.9%) and 7 (8.6%), respectively. The presence of *EGFR* mutations, *ALK* rearrangements, or *KRAS* mutations was mandatory in 12 (14.8%), 8 (9.9%), and one (1.2%) trials, respectively. Pharmaceutical companies sponsored 65 (80.2%) RCTs. Placebo was the control arm of 26 (32.1%) RCTs. The results of the trial were positive in 57 (70.4%) cases. OS and PFS were primary endpoints in 40 (49.4%) and 48 (59.3%) trials, respectively. In 9 (11.1%) RCTs, OS and PFS were co-primary endpoints. The most frequently used questionnaires to measure QoL outcomes were the European Organisation for Research and Treatment of Cancer (EORTC QLQ-C30/LC-13, *n* = 48, 59.3%), EuroQoL (EQ, various versions, *n* = 37, 45.7%), Lung Cancer Symptom Scale (LCSS, *n* = 17, 21.0%), and Functional Assessment of Cancer Therapy (FACT, various version, *n* = 13, 16.1%). Finally, we found that only 56/81 (69.1%) trials disclosed QoL results in primary publications.

**Table 1:** Characteristics of the phase III RCTs included in the analysis

	**Number**	**Percent**
**Total**	81	100
**Year of primary publication**		
**2012**	14	17.3
**2013**	8	9.9
**2014**	6	7.4
**2015**	12	14.8
**2016**	4	4.9
**2017**	9	11.1
**2018**	7	8.6
**2019**	5	6.2
**2020**	6	7.4
**2021**	10	12.3
**Journal of primary publication**		
***Annals of Oncology***	6	7.4
***British Journal of Cancer***	1	1.2
***Cancer***	1	1.2
***Cancer Cell***	1	1.2
***Clinical Lung Cancer***	1	1.2
***European Journal of Cancer***	1	1.2
***JAMA***	1	1.2
***JAMA Oncology***	4	4.9
***Journal of Clinical Oncology***	13	16.1
***Journal of Thoracic Oncology***	6	7.4
***Lancet***	4	4.9
***Lancet Healthy Longevity***	1	1.2
***Lancet Oncology***	21	25.9
***Lung Cancer***	5	6.2
***New England Journal of Medicine***	15	18.5
**Class of therapy investigated**^**a**^		
**Immunotherapy**	16	19.8
**Target therapy**	50	61.7
**EGFR inhibitors**	24	29.6
**ALK inhibitors**	8	9.9
**Others**^**b**^	18	22.2
**Chemotherapy**	17	21.0
**Control arm: placebo**		
**Yes**	26	32.1
**No**	55	67.9
**Primary tumor**^**c**^		
**Non-specified histology**	37	45.7
**NSCC**	16	19.8
**SCC**	7	8.6
**Non-specified histology—*EGFR* mutations**	6	7.4
**NSCC—*EGFR* mutations**	6	7.4
**Non-specified histology—*ALK* rearrangements**	5	6.2
**NSCC—*ALK* rearrangements**	3	3.7
**Non-specified histology—*KRAS* mutations**	1	1.2
**Funding**		
**Profit**	65	80.2
**Non-profit**	16	19.8
**Study design**		
**Superiority**	75	92.6
**Non-inferiority**	6	7.4
**Masking**		
**Open-label**	26	32.1
**Blinded**	55	67.9
**Results of the trial**		
**Positive**	57	70.4
**Negative**	24	29.6
**Countries involved in the trial**		
**Two or more**	55	67.9
**Single country**	26	32.1
**Primary endpoint**^**d**^		
**OS**	40	49.4
**PFS**	48	59.3
**ORR**	1	1.2
**Safety**	1	1.2
**QOL**	1	1.2
**QoL tool used**^**e**^		
**EORTC (QLQ-C30/QLQ-LC13)**	48	59.3
**FACT (various versions)**	13	16.1
**EuroQoL (various versions)**	37	45.7
**LCSS**	17	21.0
**SILC**	2	2.5
**SQLI**	1	1.2
**NSCLC-SAQ**	1	1.2
**QoL results reported in primary publication**^**f**^		
**Yes**	56	69.1
**No**	25	30.9

^a^Categories are not mutually exclusive. Two trials included a combination of chemotherapy plus bevacizumab in the experimental arm, compared to standard chemotherapy

^b^“Others” included the following: 2 trials included a combination of chemotherapy plus bevacizumab in the experimental arm, 2 trials tested bevacizumab, 2 ramucirumab, 2 sunitinib, 2 nintedanib, 1 apatinib, 1 fruquintinib, 1 aflibercept, 1 selumetinib, 1 anlotinib, 1 veliparib, 1 vandetanib, and 1 nytroglicerin

^c^The presence of *EGFR* mutations, *ALK* rearrangements, or *KRAS* mutations was mandatory to enroll patients in the trials reported in the table

^d^Categories are not mutually exclusive. In 9 trials, co-primary endpoints were OS and PFS. In 1, PFS and safety

^e^Categories are not mutually exclusive

^f^Among 56 trials reporting QoL data in primary publications, 10 also reported QoL analysis in secondary manuscripts. Of the 25 trials non-reporting QoL results in primary publciations, 20/25 trials published QoL results in secondary manuscripts, while 5/25 RCTs disclosed QoL data at international conferences

### Evaluation of QoL and survival outcomes

Global QoL was superior in the experimental arm, compared to the control arm, in 30 (37.0%) RCTs (Table 2). A statistically significant difference in global QoL between the experimental and control arms was not found in 48 (59.3%) trials. In 3 (3.7%) trials, QoL outcomes were worse in the experimental than in the control arm. The experimental treatment was associated with improved OS in 30/81 (37.0%) or non-improved OS in 46/81 (56.8%) RCTs. Among the 30 trials with OS-positive results, in 13 cases (*n* = 13/30, 43.3%), there was a concomitant improvement in QoL. However, we did not find a statistically significant correlation between QoL and OS outcomes (*χ*^2^ = 0.81, *p* = 0.368). PFS endpoint was met in 60/81 (74.1%) trials, while 20/81 (24.7%) RCTs failed to demonstrate a statistically significant improvement in PFS for the experimental treatment. Among the 60 trials with PFS-positive results, in 26 cases (*n* = 26/60, 43.3%), there was a concomitant improvement in QoL. Moreover, among the 20 trials with PFS-negative results, in 16 cases (*n* = 16/20, 80.0%), a concomitant improvement in QoL was not found. We recorded a statistically significant correlation between superior QoL and improved PFS outcomes (*χ*^2^ = 3.93, *p* = 0.0473).

**Table 2:** Overall survival and progression-free survival outcomes in trials with QoL results

	**Quality of life in the experimental arm**			**Total**	**Statistics**
	**Superior**	**No difference**	**Inferior**		
**Overall survival**					
**Improved**	13 (43.3%)	16 (53.3%)	1 (3.3%)	30 (100%)	***χ***^**2**^** = 0.81, *p* = 0.368**^**†**^
**Non-improved**	15 (32.6%)	29 (63.0%)	2 (4.4%)	46 (100%)	
**Non-available data**	2 (40.0%)	3 (60.0%)	–	5 (100%)	
**Total**	30	48	3	81	
**Progression-free survival**					
**Improved**	26 (43.3%)	32 (53.3%)	2 (3.3%)	60 (100%)	***χ***^**2**^** = 3.93, *p* = 0.0473**^**‡**^
**Non-improved**	4 (20.0%)	15 (75.0%)	1 (5.0%)	20 (100%)	
**Non-available data**	–	1 (100%)	–	1 (100%)	
**Total**	30	48	3	81	

^†^Statistics refers to the comparison of improved overall survival versus non-improved plus non-available data

^‡^Statistics refers to the comparison of improved progression-free survival versus non-improved plus non-available data

Next, we evaluated QoL outcomes in the cohort of 81 trials based on the drug class of the experimental treatment. In 10/16 (62.5%) RCTs testing immunotherapy, global QoL was superior in the experimental arm (Additional file 2: Table S2). Furthermore, in 16/48 (33.3%) and 4/15 (26.7%) trials testing target therapies or chemotherapy, respectively, global QoL results were better in the experimental arm than in the control arm (Additional file 2: Table S2). In more detail, in 8/24 (33.3%) trials testing EGFR inhibitors and in 7/8 (87.5%) testing ALK inhibitors, global QoL outcomes were superior in the experimental arm than in the control arm (Additional file 3: Table S3).

Next, we investigated a potential correlation between QoL and OS or PFS outcomes based on drug class. The results are reported in Table 3. We did not find a statistically significant correlation between QoL outcomes and OS outcomes for any drug class (immunotherapy, target therapy, or chemotherapy). Similarly, QoL improvements were not correlated with PFS improvements in trials testing immunotherapy or chemotherapy. Instead, we found that in 16/38 (42.2%) trials testing target therapies with positive PFS results, QoL outcomes were superior in the experimental arm (*p* = 0.0196). Furthermore, a statistically significant positive correlation between QoL results and PFS outcomes was found in the 32 trials testing EGFR or ALK inhibitors (*p* = 0.0077). In more detail, in 15/35 (60.0%) trials testing EGFR or ALK inhibitors with positive PFS outcomes, global QoL results were superior in the experimental arm.

**Table 3:** Correlation of QoL results with OS and PFS outcomes, based on drug class

	**Quality of life in the experimental arm**			**Total**	***p***
	**Superior**	**No difference**	**Inferior**		
**Overall survival (immunotherapy)**					
**Improved**	8 (66.7%)	4 (33.3%)	–	12 (100%)	*0.604*
**Non-improved**	1 (50.0%)	1 (50.0%)	–	2 (100%)	
**Non-available data**	1 (50.0%)	1 (50.0%)	–	2 (100%)	
**Overall survival (target therapy)**					
**Improved**	3 (30.0%)	6 (60.0%)	1 (10.0%)	10 (100%)	> *0.99*
**Non-improved**	12 (34.2%)	22 (62.9%)	1 (2.9%)	35 (100%)	
**Non-available data**	1 (100%)	2	–	3 (100%)	
**Overall survival (chemotherapy)**					
**Improved**	2 (25.0%)	6 (75.0%)	–	8 (100%)	> *0.99*
**Non-improved**	2 (28.6%)	4 (57.1%)	1 (14.3%)	7 (100%)	
**Non-available data**	–	–	–	–	
**Overall survival (EGFR + ALK inhibitors)**^**a**^					
**Improved**	3 (37.5%)	4 (50.0%)	1 (12.5%)	8 (100%)	*0.691*
**Non-improved**	11 (47.8%)	12 (52.2%)	–	23 (100%)	
**Non-available data**	1 (100%)	–	–	1 (100%)	
**Progression-free survival (immunotherapy)**					
**Improved**	8 (66.7%)	4 (33.3%)	–	12 (100%)	*0.604*
**Non-improved**	2 (50.0%)	2 (50.0%)	–	4 (100%)	
**Non-available data**	–	–	–	–	
**Progression-free survival (target therapy)**					
**Improved**	16 (42.1%)	20 (52.6%)	2 (5.3%)	38 (100%)	*0.0196*
**Non-improved**	–	10 (100%)	–	10 (100%)	
**Non-available data**	–	–	–	–	
**Progression-free survival (chemotherapy)**					
**Improved**	2 (22.2%)	7 (77.8%)	–	9 (100%)	> *0.99*
**Non-improved**	2 (40.0%)	2 (40.0%)	1 (20.0%)	5 (100%)	
**Non-available data**	–	1 (100%)	–	1 (100%)	
**Progression-free survival (EGFR + ALK inhibitors)**^**a**^					
**Improved**	15 (60.0%)	9 (36.0%)	1 (4.0%)	25 (100%)	*0.0077*
**Non-improved**	–	7 (100%)	–	7 (100%)	
**Non-available data**	–	–	–	–	

We excluded from this analysis 2 trials including a combination of chemotherapy plus bevacizumab in the experimental arm versus chemotherapy (different from the one used in the experimental arm) alone. Fisher’s exact test was used for statistical analysis

^a^Among the 32 trials testing EGFR or ALK inhibitors, in 13 cases, target therapy alone was compared to chemotherapy; in 8 cases, target therapy was compared to target therapy; in 7 cases, target therapy plus chemotherapy was compared to chemotherapy alone; in 4 cases, target therapy was compared to best supportive care

Furthermore, we assessed whether QoL outcomes correlated with the results of the trials. We found that experimental treatments led to superior QoL in 27/57 (47.4%) trials with positive results and in 3/24 (12.5%) RCTs with negative results (*p* = 0.0028, Table 4).

**Table 4:** Correlation of QoL outcomes and trials results

	**Quality of life in the experimental arm**			**Total**	**Statistics**
	**Superior**	**No difference**	**Inferior**		
**Result of the trial**					
**Positive**	27 (47.4%)	28 (49.1%)	2 (3.5%)	57 (100%)	***p* = 0.0028﻿†**
**Negative**	3 (12.5%)	20 (83.3%)	1 (4.2%)	24 (100%)	
**Total**	30	48	3	81	

^†^Statistics refers to the comparison of positive versus negative results of the trials. “No difference” and “inferior” QoL results were summed. Statistics: Fisher’s exact test

### Assessment of QoL results description

We investigated how authors described QoL results in manuscripts. Particularly, we assessed whether QoL data were presented differently in profit versus non-profit studies. To answer this question, we only evaluated the 51 trials in which QoL outcomes were not statistically significant between the arms of the trial (*n* = 48, Table 2) or inferior in the experimental arm (*n* = 3, Table 2). Interestingly, in 11/37 (29.7%) RCTs sponsored by pharma companies, the description of QoL results was favorable (Table 5). Instead, none of the non-profit studies reported QoL data in a favorable manner (Table 5, *p* = 0.0232). In addition, we explored an association between positive studies (met primary endpoints) and a favorable description of QoL results. We found that 9/30 (30.0%) positive trials reported QoL data in a favorable fashion (Additional file 4: Table S4). Instead, only 2/21 (9.5%) negative RCTs described QoL results in a favorable manner (Additional file 4: Table S4, *p* = 0.097).

**Table 5:** Description of QoL results based on sponsorship

RCTs with no difference in QoL or inferior QoL in experimental arm (*N* = 51)	Description of QoL results		*p*
**Sponsorship**	**Neutral, coherent with results**	**Favorable**	**0.0232**
**Profit**	**26**	**11**	
**No profit**	**14**	**0**	

The analysis included only the 51 trials in which a non-statistically significant difference in QoL results was found between the experimental and control arms (*n* = 48) or with QoL outcomes inferior in the experimental arm (*n* = 3). Fisher’s exact test

## Discussion

In the last years, the prognosis of patients with metastatic NSCLC has certainly improved. Indeed, target therapies for oncogene-addicted NSCLC, as well as immunotherapy in non-oncogene-addicted tumors, have produced a remarkable extension of survival, in comparison with the previously available standard treatments. However, despite this considerable improvement in survival outcomes, less information is available about the effect of novel treatments on QoL. Previous publications revealed that new oncology therapies approved by the Food and Drug Administration (FDA) and European Medicines Agency (EMA) often lack published QoL data [2, 5]. Furthermore, only a small fraction of FDA- and EMA-approved anticancer drugs demonstrated significant improvements in QoL, compared to the standard of care [21, 22]. Herein, we investigated the effects of new treatments on QoL, in RCTs testing novel therapies in metastatic NSCLC published in a 10-year time span (2012–2021). Furthermore, we assessed whether QoL results correlated with survival outcomes.

First, we observed a high rate (*n* = 40/158, 25.3%) of trials non-including QoL assessment among study endpoints (Fig. 1). In addition, 37/158 (23.4%) RCTs, although declaring assessment of QoL among endpoints in methods and/or in the study protocol, did not report QoL data in primary or secondary publications or at international conferences (Fig. 1). These data are consistent with previous studies revealing an inadequate assessment and reporting of QoL data in RCTs in lung cancer [17, 18].

We found a positive correlation between QoL results and PFS outcomes (Table 2). Instead, a positive correlation between QoL and OS outcomes was not found (Table 2). Several reasons may explain this difference. First, we observed that 30/60 (50%) trials with positive PFS results did not register concomitant OS improvements with the experimental treatment (Table 2). Discordance between PFS and OS was previously reported in trials of NSCLC, as well as other solid tumors [3, 23, 24]. The effects of patients’ crossover in RCTs, as well as the different treatments used after the failure of experimental therapy in the trial, may certainly influence the OS results [25]. However, we think that results from this analysis should generate further confrontation and discussion in communities of clinicians and scientists. Indeed, novel treatments leading to longer OS but without concomitant improvements in QoL should be carefully evaluated by regulatory agencies. This is particularly relevant for treatments that guarantee only modest prolongation of OS.

Interestingly, we found that the correlation between QoL improvement and PFS was statistically significant only for target therapies, particularly for EGFR and ALK inhibitors (Table 3). We believe that the size of this correlation could be even larger than what we observed. Indeed, our analysis included some of the first trials with EGFR inhibitors that enrolled patients regardless of *EGFR* mutational status. The correlation between QoL improvements and PFS prolongation is probably due to the well-known efficacy of EGFR and ALK TKIs in NSCLC harboring *EGFR* mutations or *ALK* rearrangements. In addition, many of these drugs have better tolerability than chemotherapy, thus influencing the results reported in QoL questionnaires by the patients. We believe that these results may help clinicians in predicting response and treatment adherence. Indeed, an early improvement in QoL, which can be easily measured in clinical practice with QoL questionnaires, may be useful to predict response to treatments. Hence, we believe that an accurate assessment of the patients’ symptoms, as well as evaluation of QoL using specific questionnaires, should be routinely performed in clinical practice, in order to identify patients who are benefitting from treatment with target therapies and, more importantly, to recognize signs and symptoms evocative of treatment failure and disease progression. Future studies investigating a correlation between changes in QoL at early time points and response to treatment may further elucidate a predictive role for QoL questionnaires.

In our study, we evaluated the experimental treatments as “superior” in terms of QoL only when they were directly compared to the control arm. Indeed, assuming that novel treatments should guarantee an improved QoL compared to the standard of care, an “intra-arm” comparison between “before” and “after” treatment is not acceptable [26]. However, trials testing novel treatments in the adjuvant setting, compared to placebo, may require further thinking. Indeed, due to the absence of macroscopic disease-causing symptoms, for novel adjuvant treatments, compared to placebo, promoting improvements in disease-free survival (DFS) and OS, a non-deterioration of QoL, instead of real improvement, could be acceptable.

We observed that several publications reported QoL results in a favorable manner. As also previously reported, there is a correlation between sponsorship by pharma companies and favorable interpretation of QoL results (Table 5) [12]. Furthermore, we found that a favorable description of QoL results was frequent in trials with positive results (Additional file 4: Table S4). In this context, the various and non-uniform ways of reporting QoL data in manuscripts are certainly one of the causes of such misinterpretation. We found that several publications, in the description of QoL results, only reported details about improvements in lung cancer symptoms, such as cough, pain, and dyspnea, or specific functions. However, the same authors did not provide a concomitant description of GHS/global QoL, even if the main or supplementary tables of the manuscript reported a non-improvement in GHS/global QoL, hindering an appropriate and comprehensive evaluation of QoL results.

Assessment of survival outcomes, such as PFS and OS, relies on well-established methods. Instead, several different tools are used to assess QoL in trials and clinical practice. The absence of a uniformly accepted methodology impedes a homologous and systematic assessment of QoL in clinical trials, as well as a comprehensive evaluation of the effects of novel treatments in NSCLC. We found that EORTC, EuroQoL, LCSS, and FACT were the most used tools among the 81 selected RCTs (Table 1), assessing different aspects of the health-related QoL. Of note, in some cases, these tests were only listed among exploratory endpoints (data not shown), thus lacking a pre-specified hypothesis [26]. Hence, the results from these analyses should be used with caution, particularly in terms of statistical evaluation.

We acknowledge that our work has some limitations. First, the data regarding the correlation between QoL results and PFS/OS outcomes are influenced by the fact that a large fraction of RCTs (*n* = 77/158, 48.7%) did not disclose QoL results. Therefore, our analysis was limited to only 81 RCTs, thus influencing a global evaluation in all trials of metastatic NSCLC. Next, even in studies reporting QoL data, during the course of the trial, a variable percentage of patients did not compile QoL tests, further reducing the amount of available data for a correct assessment. Next, it has to be considered that the open-label design of trials may influence the way patients perceive the treatment, ultimately affecting the compilation of QoL questionnaires. Furthermore, trials included in our analysis were heterogeneous, including patients with various NSCLC histologies, different lines (first, second, or later line) of treatment, and varied tumor mutational status. However, the results from our study might stimulate further investigation in the field of QoL in NSCLC as well as other solid cancers.

## Conclusions

Our work reveals that in trials of novel systemic treatments in patients with metastatic NSCLC, improvements in QoL correlate with PFS advantage, particularly for EGFR and ALK inhibitors. Further collaboration between academic institutions and industries is needed to improve the assessment methodologies of QoL in clinical trials, as well as the way the results are reported.

## Supplementary Information

**Additional file 1: Table S1.** Characteristics of PubMed research and list of trials included in the analysis.

**Additional file 2: Table S2.** Distribution of QoL results in RCTs by drug class. We excluded from this analysis 2 trials including combination of chemotherapy plus bevacizumab in the experimental arm, versus chemotherapyalone.

**Additional file 3: Table S3.** QoL results in RCTs by target therapies.

**Additional file 4: Table S4.** Description of QoL results based on results of the trial. The analysis included only the 51 trials in which a non-statistically significant difference in QoL results was found between experimental or control armor with QoL outcomes inferior in the experimental arm. Fisher’s exact test.

## Data Availability

The data underlying this article are available in the article and in its online supplementary material. For any other request for data related to the manuscript, please contact the corresponding author.
