# Supplementary material for: Analysis of phase III clinical trials in metastatic NSCLC to assess the correlation between QoL results and survival outcomes
Source: BMC Med. 2023 Jul 3;21:234. doi: 10.1186/s12916-023-02953-0 (PMC10318754; doi:10.1186/s12916-023-02953-0)
Supplement: Supplementary file 2 — Additional file 2: Table S2. Distribution of QoL results in RCTs by drug class. We excluded from this analysis 2 trials including combination of chemotherapy plus bevacizumab in the experimental arm, versus chemotherapyalone. [file 12916_2023_2953_MOESM2_ESM.docx]

|  | Quality of life in the experimental arm | | | Total |
| --- | --- | --- | --- | --- |
|  | **Superior** | **No difference** | **Inferior** |  |
| Drug class  Immunotherapy  Target therapy  Chemotherapy | 10  (62.5%)  16  (33.3%)  4  (26.7%) | 6  (37.5%)  30  (62.5%)  10  (66.7%) | -  2  (4.2%)  1  (6.6%) | 16  (100%)  48  (100%)  15  (100%) |
| Total | 30 | 46 | 3 | 79 |

**Table S2. Distribution of QoL results in RCTs by drug class.**

We excluded from this analysis 2 trials including combination of chemotherapy plus bevacizumab in the experimental arm, versus chemotherapy (different from the one used in the experimental arm) alone.
