# Supplementary material for: Analysis of phase III clinical trials in metastatic NSCLC to assess the correlation between QoL results and survival outcomes
Source: BMC Med. 2023 Jul 3;21:234. doi: 10.1186/s12916-023-02953-0 (PMC10318754; doi:10.1186/s12916-023-02953-0)
Supplement: Supplementary file 3 — Additional file 3: Table S3. QoL results in RCTs by target therapies. [file 12916_2023_2953_MOESM3_ESM.docx]

|  | Quality of life in the experimental arm | | |
| --- | --- | --- | --- |
| Target therapy | **Superior** | **No difference** | **Inferior** |
| EGFR inhibitors  ALK inhibitors | 8  7 | 15  1 | 1  - |

**Table S3. QoL results in RCTs by target therapies.**
