# Supplementary material for: Analysis of phase III clinical trials in metastatic NSCLC to assess the correlation between QoL results and survival outcomes
Source: BMC Med. 2023 Jul 3;21:234. doi: 10.1186/s12916-023-02953-0 (PMC10318754; doi:10.1186/s12916-023-02953-0)
Supplement: Supplementary file 4 — Additional file 4: Table S4. Description of QoL results based on results of the trial. The analysis included only the 51 trials in which a non-statistically significant difference in QoL results was found between experimental or control armor with QoL outcomes inferior in the experimental arm. Fisher’s exact test. [file 12916_2023_2953_MOESM4_ESM.docx]

| RCTs with no difference in QoL or inferior QoL in experimental arm (N=51) | Description of QoL results | | *p* |
| --- | --- | --- | --- |
| Results of the trial | **Neutral, coherent with results** | **Favorable** | **0.097** |
| Positive  Negative | **21**  **19** | **9**  **2** |  |

**Table S4: Description of QoL results based on results of the trial.**

The analysis included only the 51 trials in which a non-statistically significant difference in QoL results was found between experimental or control arm (n=48) or with QoL outcomes inferior in the experimental arm (n=3). Fisher’s exact test**.**
